# Genotyping crossing parents and family bulks can facilitate cost-efficient genomic prediction strategies in small-scale line breeding programs

**DOI:** 10.1007/s00122-021-03794-2

**Published:** 2021-02-27

**Authors:** Sebastian Michel, Franziska Löschenberger, Christian Ametz, Hermann Bürstmayr

**Affiliations:** 1 Department of Agrobiotechnology, IFA-Tulln, University of Natural Resources and Life Sciences Vienna, Konrad-Lorenz-Str. 20, 3430 Tulln, Austria; 2 Saatzucht Donau GesmbH. & CoKG, Saatzuchtstrasse 11, 2301 Probstdorf, Austria

## Abstract

**Key message:**

Genomic relationship matrices based on mid-parent and family bulk genotypes represent cost-efficient alternatives to full genomic prediction approaches with individually genotyped early generation selection candidates.

**Abstract:**

The routine usage of genomic selection for improving line varieties has gained an increasing popularity in recent years. Harnessing the benefits of this approach can, however, be too costly for many small-scale breeding programs, as in most genomic breeding strategies several hundred or even thousands of lines have to be genotyped each year. The aim of this study was thus to compare a full genomic prediction strategy using individually genotyped selection candidates with genomic predictions based on genotypes obtained from pooled DNA of progeny families as well as genotypes inferred from crossing parents. A population of 722 wheat lines representing 63 families tested in more than 100 multi-environment trials during 2010–2019 was for this purpose employed to conduct an empirical study, which was supplemented by a simulation with genotypic data from further 3855 lines. A similar or higher prediction ability was achieved for grain yield, protein yield, and the protein content when using mid-parent or family bulk genotypes in comparison with pedigree selection in the empirical across family prediction scenario. The difference of these methods with a full genomic prediction strategy became furthermore marginal if pre-existing phenotypic data of the selection candidates was already available. Similar observations were made in the simulation, where the usage of individually genotyped lines or family bulks was generally preferable with smaller family sizes. The proposed methods can thus be regarded as alternatives to full genomic or pedigree selection strategies, especially when pedigree information is limited like in the exchange of germplasm between breeding programs.

**Supplementary Information:**

The online version contains supplementary material available at 10.1007/s00122-021-03794-2.

## Introduction

Genomic prediction has been postulated as a new paradigm in plant breeding several years ago, and since then implemented in several line breeding programs world-wide (Juliana et al. 2019; Borrenpohl et al. 2020; Tsai et al. 2020). Advanced and young generation lines are for this purpose usually genotyped at several thousand marker loci to elucidate the genetic relationship between them (Crossa et al. 2017). Based on these common molecular markers, the advanced generation and well-phenotyped lines are subsequently used to predict genomic breeding values for young selection candidates, for which non- or only limited phenotypic information is available yet (Heslot et al. 2012; Robertsen et al. 2019). The usage of these predicted breeding values for conducting a genomic selection is oftentimes realized during a phase of preliminary yield trials (Borrenpohl et al. 2020; Tsai et al. 2020), as the budget of larger breeding programs allows to genotype all lines entering this testing stage. Nevertheless, the incurring costs to genotype several hundred or even thousands of lines each year can be a major restriction in small-scale line breeding programs both in developed and developing countries, despite strategies like shallow or targeted genotyping-by-sequencing (Gorjanc et al. 2017; Abed et al. 2018; e Sousa et al. 2019). A classical alternative to genotyping is given by the systematic usage of pedigree records for predicting breeding values, which makes, however, the major assumption of equal parental contributions and models only the expected mean of each given progeny population. Legarra et al. (2009) as well as Christensen and Lund (2010) suggested thus a combination of both mentioned strategies by utilizing pedigree records of all individuals in the training and selection population for computing a combined relationship matrix of genotyped and non-genotyped individuals. Originally proposed for animal breeding, this single-step genomic selection method showed also some merit to increase the prediction accuracy for both genotyped and non-genotyped individuals in plant breeding contexts when enlarging the training population by non-genotyped individuals (Ashraf et al. 2016; Imai et al. 2019).

The expected population mean of a bi-parental population of non-genotyped individuals can, alternatively to pedigree records, also be inferred from the molecular marker genotypes of both parents. The referred mid-parent genotype is, e.g., frequently used in genomic hybrid breeding to construct two-way or three-way hybrids from their inbred parents (Zhao et al. 2013). In breeding programs for inbred cereals, which use multiple steps of selfing or direct derivation of homozygous material by double haploid technology, lines are pre-selected and pass through several bottlenecks before their grain yield potential is firstly tested in observation or preliminary yield trials, i.e., the stage at which genotyping is usually conducted in genomic line breeding. This issue can lead to deviations of the expected allele frequency within families, as some alleles are being fixed or distorted towards favorable alleles by breeder’s selection with respect to easy and early to assess traits like anthesis date or by genetic hitchhiking. One convenient option to address this issue is the pooling of DNA samples from multiple individuals and fingerprinting family bulks instead of employing the expected mid-parent genotype of the selection candidates. A fraction of the genotyping budget is moreover necessary with both the mid-parent or family bulk methods in comparison with a full genomic breeding strategy that requires fingerprinting all individual early generation lines, as usually the number of families and their parents is substantially smaller than the total number of these early generation lines tested in preliminary yield trials. The aim of this study was thus to compare classical pedigree with genomic prediction models based on mid-parent, family bulk, and individual genotypes as well as a single-step genomic prediction and assess their potential for small-scale line breeding programs with a limited genotyping budget.

## Materials and methods

### Plant material

A population of 4577 *F*_4:6_, *F*_5:7_ or double haploid winter wheat (*Triticum aestivum* L.) breeding lines from 1463 different families was analyzed in this study. All lines were developed and tested in the framework of variety development in the winter wheat breeding program of Saatzucht Donau GesmbH. & CoKG. The size of each family varied between 1 and 51 lines with an average of three lines per family and a genealogy of 1062 ancestors tracing back up to 13 generations. DNA from all breeding lines was extracted using a modification of the protocol outlined by Saghai-Maroof et al. (1984), and shipped in 96-well microtiter plates to Australia for genotyping with the DArTseq genotyping-by-sequencing (GBS) approach (Diversity Arrays Technology Pty Ltd 2020a). DNA samples were processed in digestion/ligation reactions using a combination of *PstI* and *HpaII* restriction enzymes similar to Kilian et al. (2012), but using two different adapters corresponding to two different restriction enzyme overhangs instead of one *PstI*-compatible adapter. Mixed *PstI–HpaII* fragments were effectively amplified in 30 rounds of polymerase chain reaction (PCR). Following the PCR, 77 cycles of single read sequencing on Illumina Hiseq2500 were run using the amplification products from each sample of the 96-well microtiter plates. The proprietary Diversity Arrays Technology Pty Ltd analytical pipelines, offered as part of the genotyping service, were used for processing the sequences generated from each of the lanes. Filtering of the raw sequences was performed for the barcoded region and the entire read. A Phred score of ≥ 30 was utilized for the barcoded region (99.9% base call accuracy with a ≥ 75% pass percentage), while for the whole read the Phred score was set to ≥ 10 (90% base call accuracy with a ≥ 50% pass percentage). Approximately 1,200,000–2,500,000 sequences per sample were utilized in marker calling and identical sequences were collapsed into FASTQCOL files. The sequences were subsequently clustered by similarity with threshold sequence distance of three base pairs. The clusters were then parsed into SNP markers using an algorithm that was trained with data of several hundred wheat populations generated on the employed genotyping-by-sequencing platform.

Quality control was applied by filtering out markers with more than 10% of missing data and a minor allele frequency smaller than 5%. One marker from completely identical marker pairs was retained at random to remove redundant markers. This resulted in a set of 2151 SNP markers with known genetic positions on the wheat consensus map v4.0 provided by Diversity Arrays Technology Pty Ltd (2020b). This map was used to generate virtual progeny populations in a simulation study involving all 4577 breeding lines as will be described below. The markers were coded as + 1 for the homozygous major and − 1 for the homozygous minor allele at a given locus, while 0 designated heterozygous loci. Phenotypic data for protein content (%) and grain yield (dt ha^−1^) from a subset of 722 lines was furthermore analyzed to conduct an empirical study. This subset comprised 63 families with an average size of 11 lines, which were chosen as at least four lines were phenotyped in several series of multi-environment trials between 2010 and 2019 from each of these families. Since molecular markers with known and unknown map position were employed in the empirical study, the utilized marker set was with 2780 SNPs slightly larger in comparison with the simulation study. The larger marker set was also used to investigate the population structure among all 4577 lines by a principal component analysis (Suppl. Figure S1). The genetic relationship among the 722 used in the empirical study was also inspected with a relationship matrix based on pedigree records (Henderson 1976) (Suppl. Figure S2).

### Statistical analysis of the phenotypic data

Phenotypic data for protein content and grain yield was assessed in 151 Central and Eastern European multi-environment trials from 2010 to 2019, which were firstly analyzed individually with various models correcting for spatial trends. Adjustments for spatial trends within each multi-environment trial were done by testing all 15 possible combinations of random row and/or column effects with/without modelling autoregressive variance–covariance structures between the plots either in row, in column or in both directions (Burgueño et al. 2000) and choosing the best fitting model by Akaike’s information criterion (AIC). The repeatability of each trial was assessed by:

1 $${h}^{2}={\sigma }_{\mathrm{G}}^{2}/\left({\sigma }_{\mathrm{G}}^{2}+\frac{1}{2}\mathrm{MVD}\right)$$

where ${\sigma }_{\mathrm{G}}^{2}$ designates the genetic variance of the investigated trait, and $\mathrm{MVD}$ the mean variance of a difference between the Best Linear Unbiased Estimates (BLUEs) (Schmidt et al. 2019). BLUEs from trials with ${h}^{2}>0.2$ were subsequently used for an across-trial analysis within each year with a linear mixed model of the form:

2 $${y}_{ij}=\mu + {g}_{i}+{t}_{j}+{e}_{ij}$$

where ${y}_{ij}$ are the BLUEs from the individual trials for each trait respectively, $\mu$ is the grand mean, and ${g}_{i}$ is the effect of the *i*^*h*^ line that was fixed to compute across-trial BLUEs. The effect of the *j*th trial ${t}_{j}$ was fixed, while the random effect ${e}_{ij}$ that incorporated both the genotype-by-environment interaction and the residual effect followed a normal distribution with $\mathbf{e} \sim { N}(0, \mathbf{I}{\sigma }_{\mathrm{e}}^{2})$. An analysis across all years for the subset of 722 lines as well as an additional set of 146 lines, which facilitated a closer connection between the years, was conducted with the linear mixed model:

3 $${y}_{ik}= \mu + {g}_{i}+{j}_{k}+{e}_{ik}$$

where ${y}_{ik}$ are the across-trial BLUEs for each trait respectively and ${j}_{k}$ designated the random year effect with $\mathbf{j} \sim { N}(0, \mathbf{I}{\sigma }_{\mathrm{j}}^{2})$, while all other previous definitions are being retained. The protein yield (dt ha^−1^) for each line was finally computed by multiplying the BLUEs from the protein content and grain yield derived from the across-year analyses. All phenotypic analyses were conducted with the mixed model packages *sommer* (Covarrubias-Pazaran 2016) and *ASReml 3* (VSN international 2018) for R (R Core Team 2020).

### Single-trait and trait-assisted prediction models

Different single-trait genomic prediction models were tested by randomly sampling 45 families and four lines per family into a training population, and 15 different families and four lines per family into a validation population. This random sampling of 180 and 60 lines into training and validation populations was repeated 100 times, and aimed to reflect a scenario where a training population of advanced generation lines tested in multi-environment trials is used to predict the performance of young selection candidates in the preliminary yield trial stage. The used resampling scheme should, however, be seen as an approximation for such a scenario in order to empirically compare the prediction models described in the next paragraphs. This approximation was made as the available phenotypic data did not allow to build reasonable scenarios for testing a prediction across breeding cycles or cohorts with adequate family sizes. The parents of the respective families were not included into the training or validation population in the empirical study since phenotypic information was not available for all of them. Genomic estimated breeding values were obtained by fitting Best Linear Unbiased Prediction Models of the from:

4 $${y}_{i}=\mu +{g}_{i}+{e}_{i}$$

where $\mu$ is again the grand mean, ${y}_{i}$ is the phenotypic value for either the protein content, grain yield or protein yield of the *i*th line, and ${e}_{i}$ the residual effect with $\mathbf{e} \sim N(0, \mathbf{I}{\sigma }_{e}^{2})$. The effect ${g}_{i}$ of the *i*th line was modelled as random with $\mathbf{g} \sim N(0, \mathbf{A}{\sigma }_{\mathrm{G}}^{2})$ for pedigree best linear unbiased predictions (P-BLUP), where $\mathbf{A}$ was computed as suggested by Henderson (1976). Alternatively, the effect ${g}_{i}$ was modelled as $\mathbf{g} \sim N(0, \mathbf{G}{\sigma }_{\mathrm{G}}^{2})$ with the genomic relationship matrix $\mathbf{G}$ for genomic best linear unbiased predictions (G-BLUP), which was built following VanRaden (2008):

5 $$\mathbf{G}=\mathbf{W}{\mathbf{W}}^{\mathrm{T}}/2\Sigma (1-{p}_{l}){p}_{l}$$

where $\mathbf{W}$ is a centered marker matrix of the *j* lines with ${W}_{jl}= {Z}_{jl}+1-2{p}_{l}$ and ${p}_{l}$ being the allele frequency at the *l*th locus. The two described pedigree and genomic prediction models represented the baseline models, and were firstly compared with a single-step genomic prediction model (SSG-BLUP) with $\mathbf{g} \sim N(0, \mathbf{H}{\sigma }_{\mathrm{G}}^{2})$ for which the hybrid relationship matrix

6 $$\mathbf{H}=\left(\begin{array}{ll}{\mathbf{A}}_{11}-{\mathbf{A}}_{12}{\mathbf{A}}_{22}^{-1}({\mathbf{G}}_{\mathrm{w}}-{\mathbf{A}}_{22}){\mathbf{A}}_{22}^{-1}{\mathbf{A}}_{21}& {\mathbf{A}}_{12}{\mathbf{A}}_{22}^{-1}{\mathbf{G}}_{\mathrm{w}}\\ {{\mathbf{G}}_{\mathrm{w}}{\mathbf{A}}_{22}^{-1}\mathbf{A}}_{21}& {\mathbf{G}}_{\mathrm{w}}\end{array}\right)$$

was built following Legarra et al. (2009) as well as Christensen and Lund (2010). The matrix ${\mathbf{A}}_{11}$ contained the pedigree relationship between non-genotyped lines, ${\mathbf{A}}_{22}$ the pedigree relationship between genotyped lines, while ${\mathbf{A}}_{12}$ and ${\mathbf{A}}_{21}$ model the pedigree relationship between genotyped and non-genotyped lines. A breeding strategy in which merely advanced generation lines are genotyped was assumed for implementing this method in the study at hand; hence the genotyped lines in $\mathbf{H}$ referred to the 180 lines in the training population and the non-genotyped lines to the 60 lines in the validation population. The inverse of $\mathbf{H}$, which is necessary for solving the underlying mixed model equations, was given by Misztal et al. (2013):

7 $${\mathbf{H}}^{-1}={\mathbf{A}}^{-1}+\left(\begin{array}{ll}0& 0\\ 0& {\left({\alpha \mathbf{G}}_{\mathrm{adj}}+{\beta \mathbf{A}}_{22}\right)}^{-1}-{\mathbf{A}}_{22}^{-1}\end{array}\right)$$

where ${\mathbf{G}}_{\mathrm{w}}={\alpha \mathbf{G}}_{\mathrm{adj}}+{\beta \mathbf{A}}_{22}$ and the scaling factors were set to $\alpha =0.95$ and $\beta =0.05$ in order to invoke a blending of the involved pedigree and genomic relationship matrices, after adjusted the genomic relationship matrix $\mathbf{G}$ by solving:

8 $$a+b\cdot \overline{\mathrm{diag}(\mathbf{G})}=\overline{\mathrm{diag}({\mathbf{A}}_{22})}$$

9 $$a+b\cdot \bar{\mathbf{G}}=\overline{{\mathbf{A}}_{22}}$$

and setting ${\mathbf{G}}_{\mathrm{adj}}=a+b\mathbf{G}$ as suggested by Christensen et al. (2012). Alternatively, a genomic breeding strategy that, like the single-step genomic prediction, targeted the family average but without pedigree information was tested by inferring the expected genotype of each family in the validation population from the genotypes of the parents by:

10 $${\mathrm{MP}}_{\mathrm{f l}}= \frac{1}{2}({m}_{\mathrm{fl}}+{f}_{\mathrm{fl}})$$

where ${m}_{\mathrm{fl}}$ and ${f}_{\mathrm{fl}}$ are the maternal and paternal genotype calls at the *l*th locus of the *f*th family. This method rendered the expected genotype of each family essentially equivalent to the mid-parent (MP) or an *F*_1_ genotype from a bi-parental cross. The potential of using pooled DNA samples from a family bulk was furthermore tested by averaging across the marker allele calls at each locus of all individuals in such a bulk, as suggested in the animal breeding literature (Baller et al. 2020):

11 $${F}_{\mathrm{fl}}= \frac{{\sum }_{i=1}^{{n}_{\mathrm{f}}}{g}_{\mathrm{fil}}}{{n}_{\mathrm{f}}}$$

where ${g}_{\mathrm{il}}$ is the genotype of the *i*th line in the *f*th family at the *l*th locus, and ${n}_{\mathrm{f}}$ is the number of lines in the *f*th family. Equation (11) approximated thus the outcome when genotyping a bulk of individuals from a given family with a genotyping-by-sequencing-based approach or using a quantitative estimate of allele calls at each locus (Bell et al. 2017). Alternatively, the resulting average genotype calls were rounded to $+1$ if ${F}_{\mathrm{fl}}>0.5$, $-1$ if ${F}_{\mathrm{fl}}<-0.5$ and to $0$ if $-0.5<{F}_{\mathrm{fl}}<0.5$, which reflected a situation when using discrete allele calls from a SNP Array (Alexandre et al. 2019, 2020). The individual marker allele calls of each line in the validation population were finally replaced by the mid-parent and family bulk genotypes in the marker matrix $\mathbf{W}$ in order to accommodate them in the computation of the genomic relationship matrix $\mathbf{G}$ by Eq. (5):

12 $$\mathbf{G}=\left(\begin{array}{ll}{\mathbf{G}}_{11}& {\mathbf{G}}_{12}\\ {\mathbf{G}}_{21}& {\mathbf{G}}_{22}\end{array}\right)$$

where the matrix ${\mathbf{G}}_{11}$ contains the genomic relationship in the training population with individual genotyped lines, ${\mathbf{G}}_{22}$ the relationship between the lines in the validation population expressed either by their mid-parent of family bulk genotypes, whereas ${\mathbf{G}}_{12}$ and ${\mathbf{G}}_{21}$ model the genomic relationship between the lines in the training and validation population. It should be noticed that in Eq. (12) identical genotypes are contained in ${\mathbf{G}}_{22}$ and $\mathbf{G}$ is accordingly singular and cannot be inverted. Hence, the distribution of the random line effect in (4) was set to $\mathbf{g} \sim N(0, {\mathbf{G}}_{\mathrm{mod}}{\sigma }_{\mathrm{G}}^{2})$ where ${\mathbf{G}}_{\mathrm{mod}}=\alpha \mathbf{G}+\beta \mathbf{I}$**,** with $\mathbf{I}$ being an identity matrix and $\alpha =\beta =0.5$, which corresponded to blend $\mathbf{G}$ with a pedigree relationship matrix of independent non-inbred founder individuals. Given ${\mathbf{G}}_{\mathrm{mod}}$ was based on mid-parent genotypes the predictions will be referred to M-BLUP herein, whereas in cases when ${\mathbf{G}}_{\mathrm{mod}}$ was based on pooled DNA samples from family bulks the model will be designated as F-BLUP_Array-like_ or F-BLUP_GBS-like_ depending if rounded or unrounded averaged allele calls were used. A genomic and pedigree-based heritability was determined by fitting either a P-BLUP or G-BLUP model with all 722 phenotyped lines and expressed as:

13 $${h}^{2}={({\sigma }_{\mathrm{P}}^{2}-\sigma }_{\mathrm{e}}^{2})/{\sigma }_{\mathrm{p}}^{2}$$

where ${\sigma }_{{\rm P}}^{2}$ is the phenotypic variance, i.e., the unbiased sample variance computed from the across-year BLUEs of the investigated trait, and ${\sigma }_{{\rm e}}^{2}$ the residual variance estimated by model (4).

It is, however, commonly known that pedigree prediction models can only discriminate between family averages (Daetwyler et al. 2007), which is likewise a limitation of the mid-parent and family bulk genomic prediction models. Exploiting pre-existing information of correlated traits or the trait of interest per se, e.g., from observation trials can circumvent this disadvantage (Pszczola et al. 2013; Michel et al. 2020a). Prediction models with individual or whole-family genotypic information of the validation population were thus tested for the target trait protein yield, where either the protein content or grain yield was exploited as pre-existing sources of phenotypic information or secondary traits for an indirect selection. The above-described Best Linear Unbiased Prediction Models were for this purpose extended to:

14 $${y}_{i}=\mu +\gamma \cdot {x}_{i}+{g}_{i}+{e}_{i}$$

where $\mu$ is again the grand mean, ${g}_{i}$ the random effect of the of the *i*th line, and ${y}_{i}$ are the phenotypic values, i.e., across-year BLUEs of the protein yield, while $\gamma$ is the regression coefficient for the covariate ${x}_{i}$ containing either the phenotypes for protein content or grain yield. The genomic estimated breeding values of the *i*th line were accordingly computed by

15 $${\mathrm{GEBV}}_{i}=\mu +\widehat{\gamma }\cdot {x}_{i}+{\widehat{g}}_{i}$$

where $\widehat{\upgamma }$ and ${\widehat{g}}_{i}$ are the estimate of the regression coefficient and the predicted line performance respectively, while ${\mathrm{x}}_{\mathrm{i}}$ is in this case the observed phenotype for protein content or grain yield of the *i*th line in the validation population. The resampling for these trait-assisted prediction models was likewise repeated 100 times and compared with utilizing solely the protein content or grain yield as predictor traits for an indirect selection. The prediction ability for all models was assessed by correlating the predicted with the observed phenotypic performance. The relative selection gain was computed as the relative difference between a fraction of the best 20–80% of the lines based on each of the tested prediction and the phenotypic average of the entire validation population, while pairwise comparisons between observed and all predicted performance values were made and expressed as the percentage of co-selected lines. The pedigree and hybrid relationship matrices, $\mathbf{A}$ and $\mathbf{H}$, were generated with the R package *AGHmatrix* (Amadeu et al. 2016) and all prediction models were fitted with *sommer* (Covarrubias-Pazaran 2016).

### Simulation and selection among virtual populations

The above-described models were subsequently investigated in an additional simulation study, where bi-parental families actually tested in preliminary yield trials in 2014, 2015, 2016, 2017 or 2018 were 50 times randomly sampled and the marker data from their parents used to simulate virtual progeny populations similar to Bernardo (2014) and Mohammadi et al. (2015). A total of 30 families were sampled for each of the mentioned years and 90 progenies were simulated per family with the *R/qtl* package (Broman et al. 2003) based on a random sample of 2100 marker loci with known genetic map position and the marker genotypes of their parents. These virtual progeny populations constituted the validation population in the simulations, while 180 additional lines with observed marker data were sampled into a training population. The sampling of the training population was thereby restricted to lines that were tested in years preceding 2014, 2015, 2016, 2017 or 2018, respectively, and included either the parents of the sampled families or explicitly excluded them from the sampling process. It should be noticed that the random sampling was restricted to a number of $n=180-p$ lines when all $p$ parents were included in the training population. Hence, the simulation study aimed to reflect a scenario where a training population of advanced generation lines from previous breeding cycles or cohorts is used to predict the performance of another cohort comprised of young selection candidates in the stage of preliminary yield trials.

A quantitatively inherited trait was simulated by sampling ${N}_{\mathrm{QTL}}=100$ marker loci, while the other set of ${N}_{\mathrm{SNP}}=2000$ markers represented linked loci for building genomic relationship matrices. QTL effects were randomly sampled from ${\varvec{\upalpha}} \sim N(0, 1)$ to compute estimated breeding values by:

16 $${\mathbf{P}}_{\mathbf{e}\mathbf{b}\mathbf{v}}=\mathbf{Q}{\varvec{\upalpha}}+\mathbf{e}={\mathbf{P}}_{\mathbf{t}\mathbf{b}\mathbf{v}}+\mathbf{e}$$

where marker genotypes of lines in the training and validation population are contained in the matrix $\mathbf{Q}$, and ${\varvec{\upalpha}}$ is a vector of QTL effects that were used to derive the vector ${\mathbf{P}}_{\mathbf{t}\mathbf{b}\mathbf{v}}$ of true breeding values. The entries for the vector of error effects $\mathbf{e}$ were randomly sampled from a normal distribution with zero mean and a variance equal to:

17 $${\sigma }_{e}^{2}= {\sigma }_{\mathrm{tbv}}^{2} \times \left( \frac{1-{h}^{2}}{{h}^{2}} \right)$$

where ${\sigma }_{\mathrm{tbv}}^{2}$ is the variance of the true breeding values, and ${h}^{2}$ an aspired entry-mean heritability of ${h}^{2}=0.30$, ${h}^{2}=0.50$, and ${h}^{2}=0.70$ that was separately determined for each training population. The corresponding entry-mean heritability of the validation population was on the other hand set to ${h}^{2}=0.10$, ${h}^{2}=0.30$, and ${h}^{2}=0.50$ in order to reflect the lower accuracy of testing in preliminary yield trials. The actual selection candidates were thereafter determined by sampling a set of 300 lines from the entire validation population of 2700 lines or, according to their true breeding value, among the best 30–90% of these lines (validation scheme A). The latter was done to examine the effect of conducting a selection before actually genotyping lines in early generations. Alternatively, the population of selection candidates was built by randomly and equally sampling 1–5, 10, 20, or 40 lines from each of the families constituting the entire validation population of 2700 lines in order to investigate the influence of the family size on the efficiency of the tested prediction models (validation scheme B).

All prediction models were firstly tested without taking advantage of pre-existing information of the selection candidates. Assuming that such pre-existing information of the selection candidates and a trait of interest per se is in some cases already available in the stage of preliminary yield trials, the tested prediction models were extended by a group effect separating the training and validation population when exploiting this information source:

18 $${y}_{hi}=\mu +{s}_{h}+{g}_{hi}+{e}_{hi}$$

where $\mu$ is again the grand mean, ${y}_{hi}$ is the estimated breeding value of the *i*th line within the *h*th group, ${s}_{h}$ is the fixed group effect separating the training and validation population, and ${g}_{hi}$ is the random line effect with $\mathbf{g} \sim N(0, \mathbf{A}{\sigma }_{\mathrm{G}}^{2})$ for pedigree, $\mathbf{g} \sim N(0, \mathbf{G}{\sigma }_{\mathrm{G}}^{2})$ for genomic predictions based of individual genotypes, $\mathbf{g} \sim N(0, \mathbf{H}{\sigma }_{\mathrm{G}}^{2})$ for single-step genomic predictions, and $\mathbf{g} \sim N(0, {\mathbf{G}}_{\mathrm{mod}}{\sigma }_{\mathrm{G}}^{2})$ for genomic predictions based of mid-parent or family bulk genotypes. Heterogenous residual variances were moreover modelled in (18) for each group to take the different phenotypic data quality for the training population and the pre-existing information of the selection candidates into account. The accuracy of the respective prediction models was finally assessed by correlating the predicted with the true breeding values. A simulated dataset and accompanied R Code are being available as supplementary material to illustrate the utilized models.

## Results

The estimated genomic heritability was medium to high as expected for grain yield and protein content in an unbalanced dataset across multiple locations and years (Table 1). The correlation between protein content and grain yield was negative (*r* = −0.44), while a positive correlation was observed between protein yield and protein content (*r* = 0.39) as well as protein yield and grain yield (*r* = 0.65). The heritability of the protein yield was furthermore slightly higher than for grain yield, most likely as the former was computed as a multiplicative index from the estimated means of the component traits.

**Table 1:** Mean, range, and heritability for grain yield, protein content, and protein yield for the population of 722 lines involved in the empirical study

Trait	Min	Mean	Max	${h}_{\mathrm{GBLUP}}^{2}$^a^	${h}_{\mathrm{PBLUP}}^{2}$^b^
Grain yield (dt ha^−1^)	53.5	68.6	82.0	0.41	0.60
Protein content (%)	11.7	14.0	16.3	0.68	0.69
Protein yield (dt ha^−1^)	7.7	9.6	11.5	0.49	0.68

^a^Heritability based on a Genomic Best Linear Unbiased Prediction model

^b^Heritability based on a Pedigree Best Linear Unbiased Prediction model

**Fig. 1:**
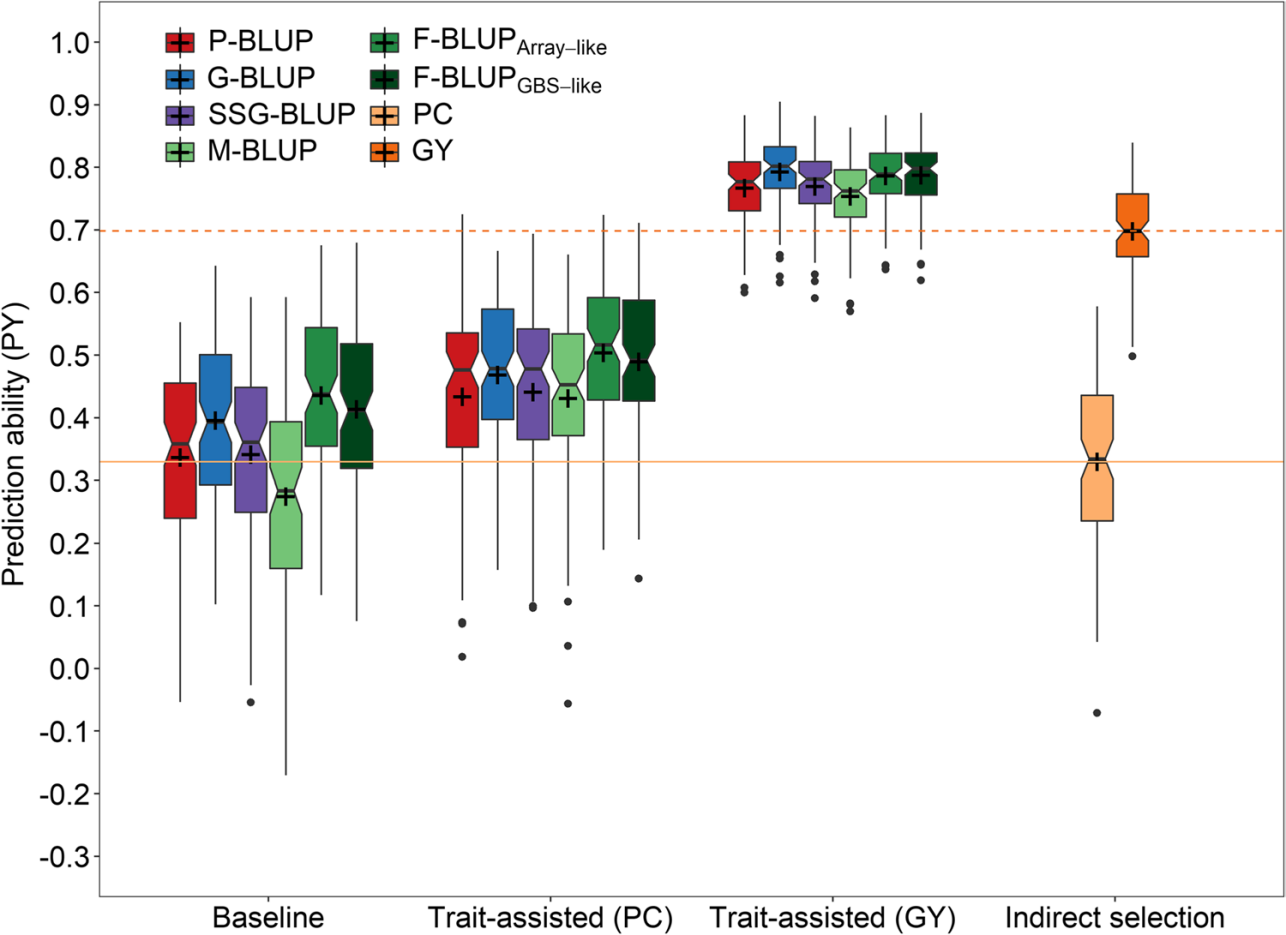
Boxplots of the prediction ability for protein yield (PY) using prediction models with pedigree relationships (P-BLUP), genomic relationships from individual genotyped lines (G-BLUP), a combined genomic-pedigree relationship matrix (SSG-BLUP) as well as mid-parent (M-BLUP) and family bulk genotypes of the selection candidates with rounded (F-BLUP_Array-like_) or unrounded (F-BLUP_GBS-like_) averaged allele calls. The respective baseline models were compared with a trait-assisted selection exploiting pre-existing information of the secondary traits protein content (PC) or grain yield (GY) as well as with an indirect phenotypic prediction by the protein content (solid horizontal line) or grain yield (dashed horizontal line)

The prediction ability for all three investigated traits was generally lower when employing a P-BLUP (*r* = 0.391) or SSG-BLUP model (*r* = 0.400) in comparison with a G-BLUP model with individual genotypes (*r* = 0.455) (Table 2). Using mid-parent genotypes in the M-BLUP model resulted in a similar performance as pedigree-based predictions (*r* = 0.391), whereas using family bulk genotypes in the F-BLUP models gave the highest average prediction ability. The difference between using rounded (F-BLUP_Array-like_) or unrounded (F-BLUP_GBS-like_) average allele calls was, however, marginal (*r*_[Array-like]_ = 0.462 vs. *r*_[GBS-like]_ = 0.457). Exploiting pre-existing data of the secondary trait protein content, i.e., a highly heritable but weakly correlated trait, to predict protein yield was generally beneficial with pedigree or genomic relationship information and resulted in higher prediction abilities than feasible by indirect phenotypic selection (Fig. 1). The same observation was made when using pre-existing information of grain yield, i.e., a low to medium heritable but strongly correlated secondary trait. The ranking of the different prediction methods followed thus in the trait-assisted prediction case likewise the pattern $\mathrm{PBLUP}\cong \mathrm{SSGBLUP}\cong \mathrm{MBLUP}<\mathrm{GBLUP}\cong \mathrm{FBLUP}$, which was also reflected by the relative selection gain and the proportion of co-selected lines (Suppl. Figures S3-S6).

**Table 2:** Average prediction abilities obtained in the 100 times replicated cross-validation scheme with the 63 families containing the 722 lines in the empirical study

Trait	P-BLUP	G-BLUP	SSG-BLUP	M-BLUP	F-BLUP_Array-like_	F-BLUP_GBS-like_
Grain yield	0.375^a^	0.413^ab^	0.390^ab^	0.439^b^	0.445^b^	0.435^b^
Protein content	0.469^ac^	0.539^b^	0.481^ac^	0.440^a^	0.508^bc^	0.515^bc^
Protein yield	0.328^a^	0.412^b^	0.330^a^	0.293^a^	0.434^b^	0.422^b^
Average	0.391^a^	0.455^b^	0.400^a^	0.391^a^	0.462^b^	0.457^b^

Prediction models involved the usage of pedigree (P-BLUP) or genomic relationships from individual genotyped lines (G-BLUP) as well as a combined relationship matrix (SSG-BLUP) and were compared with genomic relationship matrices based on mid-parent (M-BLUP) or family bulk genotypes of the selection candidates with rounded (F-BLUP_Array-like_) or unrounded (F-BLUP_GBS-like_) averaged allele calls

Prediction abilities within each row that share the same letter are not significantly different at *α* = 5%

**Fig. 2:**
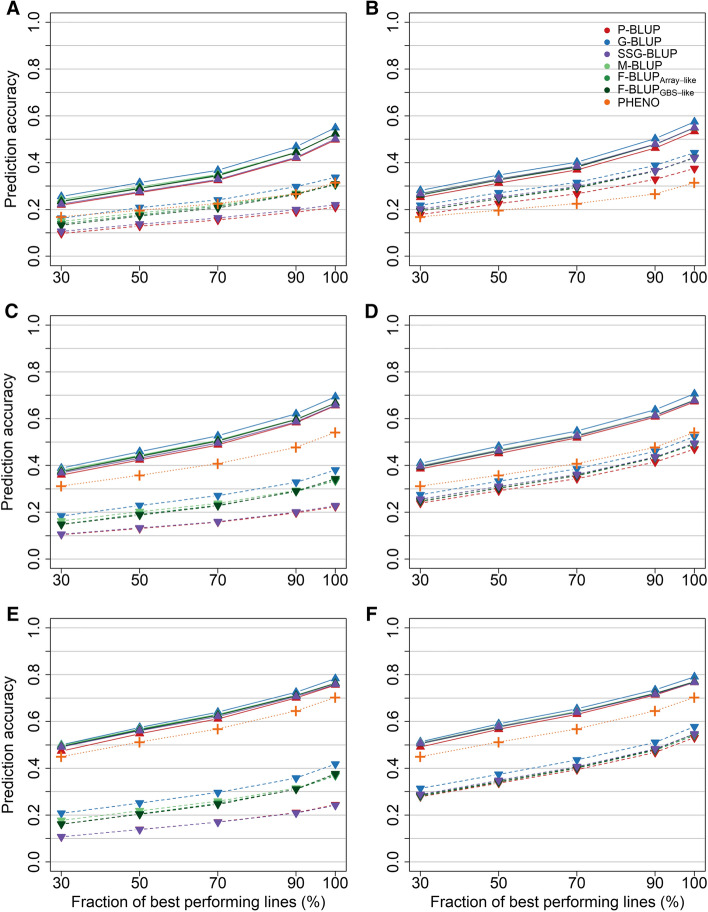
Prediction accuracy for different pre-selection intensities of the selection candidates for quantitative inherited traits with an entry-mean heritability of *h*^2^ = 0.10 (**A** + **B**), *h*^2^ = 0.30 (**C** + **D**), and *h*^2^ = 0.50 (**E** + **F**) in the simulation study (validation scheme A). The prediction models were based on pedigree (P-BLUP) or genomic relationships from individual genotyped lines (G-BLUP), a combined genomic-pedigree relationship matrix (SSG-BLUP) as well as mid-parent (M-BLUP) and family bulk genotypes of the selection candidates with rounded (F-BLUP_Array-like_) or unrounded (F-BLUP_GBS-like_) averaged allele calls, and were compared with phenotypic selection based on observed performance records (PHENO). All models were fitted with (solid lines) or without (dashed lines) exploiting pre-existing information of the selection candidates, while parents were either excluded (left column) or included (right column) in the training population

The simulation study showed on the other hand a similar prediction accuracy for the M-BLUP and F-BLUP models, which outperformed the P-BLUP and SSG-BLUP models when no pre-existing data of selection candidates was available in validation scheme A with a pre-selected fraction of selection candidates (Fig. 2, left column). All models were, however, inferior to phenotypic selection based on observed performance records with entry-mean heritabilities of *h*^2^ = 0.30 and *h*^2^ = 0.50 in this case. This disadvantage became smaller if parents were part of the training population, in which case G-BLUP was still preferable to the other prediction models (Fig. 2, right column). Given that pre-existing phenotypic data was already available, e.g., from preliminary yield trials, an advantage of modelling relationships among lines was found in comparison with a phenotypic selection based solely on observed performance, especially under the assumption of a low entry-mean heritability (*h*^2^ = 0.10). Modelling genomic relationships among individual lines resulted again in the highest prediction accuracy in this scenario, followed by the employment of family bulk and mid-parent genotypes. The relative difference between the various models was, however, marginal in this situation, ranging on average from 5.5% with *h*^2^ = 0.10 to 1.7% with *h*^2^ = 0.50. Notably, the prediction accuracy became always lower if the lines in the validation population came from a population with a stronger pre-selection towards higher average performance.

**Fig. 3:**
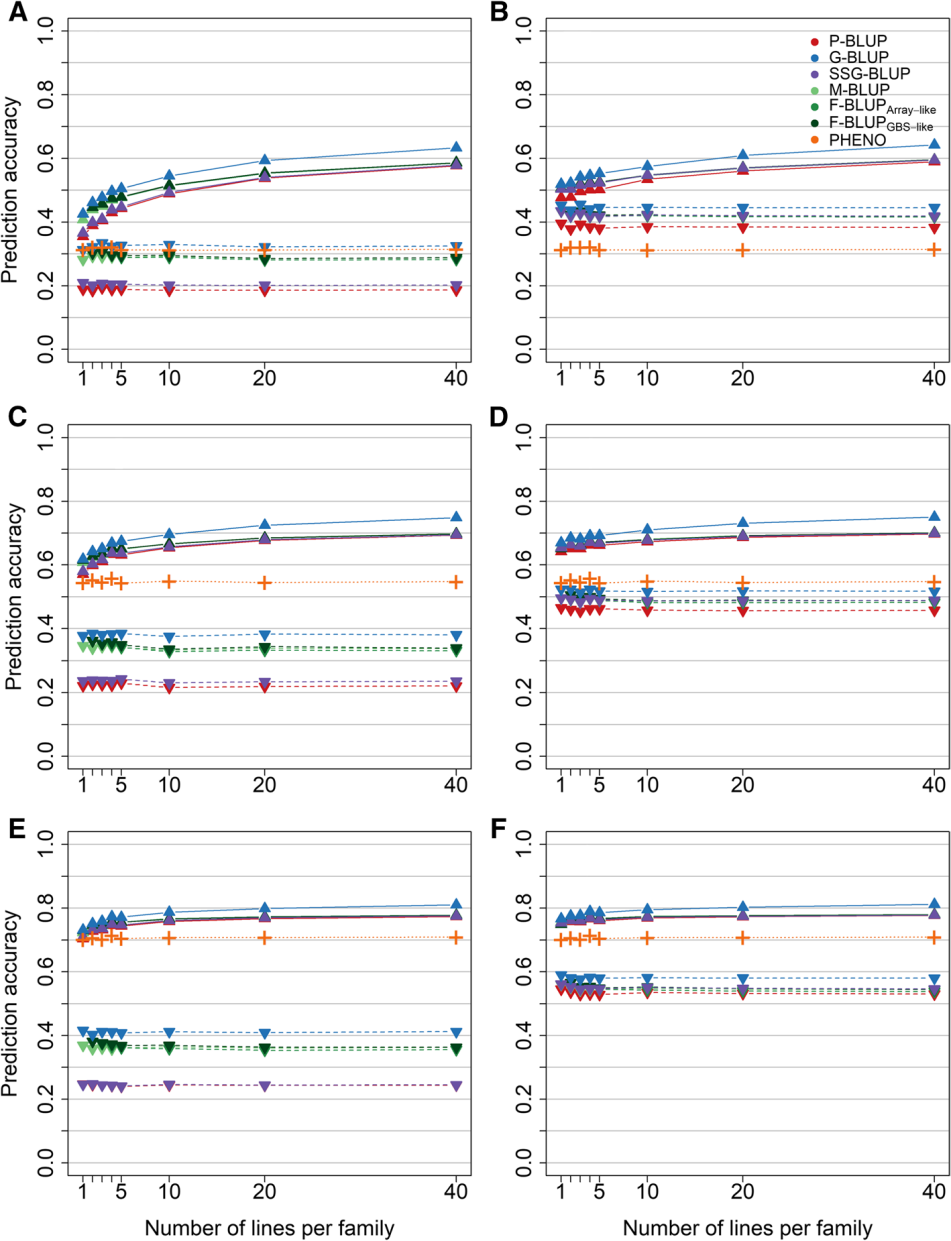
Prediction accuracy for a varying number of lines equally sampled from each family in the validation population for quantitative inherited traits with an entry-mean heritability of *h*^2^ = 0.10 (**A** + **B**), *h*^2^ = 0.30 (**C** + **D**), and *h*^2^ = 0.50 (**E** + **F**) in the simulation study (validation scheme B). The prediction models were based on pedigree (P-BLUP) or genomic relationships from individual genotyped lines (G-BLUP), a combined genomic-pedigree relationship matrix (SSG-BLUP) as well as mid-parent (M-BLUP) and family bulk genotypes of the selection candidates with rounded (F-BLUP_Array-like_) or unrounded (F-BLUP_GBS-like_) averaged allele calls, and were compared with phenotypic selection based on observed performance records (PHENO). All models were fitted with (solid lines) or without (dashed lines) exploiting pre-existing information of the selection candidates, while parents were either excluded (left column) or included (right column) in the training population

Aside from the effect of a pre-selection, the effect of the family size on the accuracy of the different prediction models was investigated by sampling an equal number of lines from each family into the validation population representing the selection candidates (validation scheme B). The prediction accuracy was again higher when the parents of each family were included in the training population (Fig. 3, right column) in comparison with a model training without phenotypic records of the parents (Fig. 3, left column). Irrespective of parental information, all prediction models were mostly invariant to the alternations in the family size when no pre-existing information of the selection candidates was exploited, although the F-BLUP models with family-bulk genotypes showed a slightly higher performance in comparison with the usage of mid-parent genotypes in the M-BLUP model for smaller family sizes. An increase in prediction accuracy was on the other hand observed when predicting larger families and at the same time utilizing pre-existing information for model training, most likely due to the inclusion of an increasing number of full-sibs into the training population. The same tendencies could also be found with the empirical data, although the differences were much subtler in this case (Suppl. Figures S7 + S8).

## Discussion

Fingerprinting individual lines in both the training and validation or selection population is probably the most common approach when implementing genomic prediction in a line breeding program. Although such a strategy resulted in the study at hand as well as other studies in a high predictive performance (Haikka et al. 2020a; Tsai et al. 2020), the additional costs might be too high for many small-scale line breeding programs in developed and developing countries. A trade-off is in these cases possibly needed, e.g., by reducing the phenotyping intensity and reallocate the available budget, which would even then not always allow to fingerprint several hundred lines each year. The usage of pedigree data invokes in theory no additional costs aside from record keeping in a database system, although missing and erroneous pedigree records are well-known issues in practical applications (Munoz et al. 2014; Endelman et al. 2017). A clear constraint of a pedigree-based selection is though given by the inability to target the Mendelian sampling term, i.e., lines within a given family receive the same breeding value and cannot be differentiated (Daetwyler et al. 2007). The Mendelian sampling can, however, be addressed in a pedigree-assisted selection by exploiting pre-existing information of correlated secondary traits or the trait of interest per se (Pszczola et al. 2013). Despite the premise that genomic prediction is oftentimes implemented at the stage of preliminary yield trials in line breeding programs, very similar prediction abilities have been found for pedigree-assisted and genomic-assisted selection when pre-existing information from such trials is included into the modeling process (Juliana et al. 2020; Michel et al. 2020b). The usage of the protein content or grain yield as secondary traits for predicting the target trait protein yield resulted exemplarily also in a smaller difference between genomic and pedigree predictions.

A further alternative in the above-mentioned prediction strategy is therefore given by using the mid-parent genotype of each family instead of pedigree records. This would moreover require to fingerprint merely a couple of dozen crossing parents instead of hundreds of selection candidates each year as in a full-scale genomic selection approach. These potential crossing parents can be employed as training population, and might comprise advanced generation well-phenotyped breeding lines from a breeding programs native gene pool as well as further germplasm of interest from other programs. Genotyping crossing parents as in the mid-parent method has moreover several conceptional advantages in comparison with the pedigree method, e.g., the option to genomically plan crosses for a more efficient diversity management within a breeding program (Osthushenrich et al. 2017; Akdemir et al. 2019; Neyhart et al. 2019). Further possibilities opened up by genotyping crossing parents are the availability of genomic predictions for difficult to phenotype traits that cannot be observed every year, like winter hardiness (Beil et al. 2019; Michel et al. 2019), or for costly to phenotype traits like baking quality (Hayes et al. 2017; Ben-Sadoun et al. 2020) and mycotoxin content (Haikka et al. 2020b; Verges et al. 2020). These predictions can be valuable inputs to further guide parental choices as phenotyping for these traits might be restricted to a few best performing advanced generation lines in small-scale breeding programs. The mentioned points pose thus a clear advantage of mid-parent genotypes in comparison with pooled DNA samples from family bulks taken in the stage of preliminary yield trials, since no individual genotypic information of potential parents is assessed in the latter. This constraint makes it necessary to re-genotype specific lines in advanced generations to build and extend a training population in this approach. However, a deviation from the expected population average with respect to parental contributions, e.g., by factors like random genetic drift and pre-selection most likely resulted in a higher prediction ability of the family bulk method in comparison with the mid-parent genotype as well as single-step genomic prediction methods (Suppl. Figure S9). This deviation was smaller for validation scheme A with different pre-selection intensities in the simulation study as compared to the empirical study. It was in this case, however, also dependent on the family size with smaller families having a larger deviation from the expectation (Suppl. Figure S10). The latter effect was also evident in validation scheme B with an equal sampling of lines from each family (Suppl. Figure S11), which indicated that the usage of individually genotyped lines or family bulks might be slightly preferable in comparison with methods that are based on expected population averages such as pedigree predictions when families are small. Combining the genomic relationship matrix of family bulks with pedigree information by blending both matrices represented beyond that also an interesting option, which did, however, not show any or a negligible advantage, except when the number of genotyped lines in the training population were four times smaller than the number of non-genotyped lines (data not shown).

One drawback of the study at hand is certainly that the expected genotyping result of such family bulks was merely computed based on genotype calls from individual lines, and that technical errors during DNA pooling like the overrepresentation of one breeding line was ignored similarly as in previous animal breeding studies (Alexandre et al. 2019, 2020; Baller et al. 2020). Although the influence of such an overrepresentation on the prediction ability has yet to be determined experimentally, it was tested in silico by increasing the proportion of one line in a given family bulk as well as increasing the number of families with an overrepresented line in order to obtain a hint of its impact (Suppl. Figure S12). Using the empirical dataset for this investigation, a decrease in prediction ability of up to 5% was observed when multiple families were biased towards the genotype of one family member. For the purpose of actual applications, it is thus recommendable to aim at bulking plant material or DNA in equal proportions with respect to the family members to appropriately reflect the families’ genetic makeup during the generation of high-quality genotypic data (Saccomanno et al. 2020).

The reliance on genotypic data to clarify relationships in breeding programs has the general benefit of modeling realized instead of expected relationships, and can also be used to clarify genetic relationships among a native gene pool and potential parents introduced into a breeding program from external sources. This might be especially valuable for germplasm acquired in the framework of the breeders’ exemption, for which detailed pedigree information is often absent. Hence, using a combination of the mid-parent and family bulk genotyping strategies can constitute an interesting alternative to pedigree selection when no systematic or merely shallow pedigree information is available. This can be particularly convenient during the first phase of implementing genomic selection in a line breeding program as seed of all crossing parents used in the past, whose progenies are currently pending selection candidates, might not be available any more due to long breeding cycles in the development of line cultivars. Genotyping advanced generation lines, crossing parents tested in more recent years as well as family bulks of current early generation selection candidates being tested in preliminary yield trials can thus be a promising starting point when initiating such an endeavor.

## Conclusions

Genotyping individual lines for a genomic-based selection showed the highest advantage when no pre-existing phenotypic data was available as, in contrast to other methods employed in this study, this strategy allowed in such cases a differentiation between lines within families. Notwithstanding, a high predictive performance was likewise achieved by models using mid-parent or family bulk genotypes in a genomic breeding strategy with pre-existing information of the selection candidates. This predictive performance can moreover be realized, depending on the breeding program, with a much smaller genotyping budget than a full genomic breeding strategy in which all individual early generation lines are genotyped. Assuming for example that 100 advanced generation breeding lines are tested in multi-environment trials each year in a typical small-sized line breeding program, it would be most likely feasible to genotype all of them in order to build and update a training population. Assuming further that the selection population of young breeding lines in such a program comprises around 1000 individuals from 50 crosses, the genotyping costs in the mid-parent or family bulk methods would amount 10% or 15% of the mentioned full genomic breeding strategy. Given the constraint of a fixed budget in a small-scale breeding program, these additional expenses might, e.g., be readily covered by reallocating phenotyping resources used for grain yield screening in a few dozen field plots. The proposed strategies can thus be regarded as alternatives to the mentioned full genomic or pedigree prediction approaches, with some conceptional benefits to the latter, like the possibility to clarify relationship with lines coming from other breeding programs, a more elaborate diversity management and consequently the genomic planning of crosses.

## Supplementary Information

Below is the link to the electronic supplementary material.

Supplementary file1 (PDF 17555 KB)

Supplementary file2 (R 10 KB)

Supplementary file3 (CSV 1735 KB)
