# Supplementary material for: Genotyping crossing parents and family bulks can facilitate cost-efficient genomic prediction strategies in small-scale line breeding programs
Source: Theor Appl Genet. 2021 Feb 27;134(5):1575–86. doi: 10.1007/s00122-021-03794-2 (PMC8081688; doi:10.1007/s00122-021-03794-2)
Supplement: Supplementary file 1 — Supplementary file1 (PDF 17555 KB) [file 122_2021_3794_MOESM1_ESM.pdf]

Supplementary material

Article Title: Genotyping crossing parents and family bulks can facilitate cost-efficient genomic prediction strategies in small-scale line breeding programs

Journal: Theoretical and Applied Genetics

Authors: Sebastian Michel, Franziska Löschenberger, Christian Ametz, Hermann Bürstmayr

Name, affiliation, and email of corresponding author:

Sebastian Michel
Department for Agrobiotechnology (IFA-Tulln)
Institute for Biotechnology in Plant Production
University of Natural Resources and Life Sciences, Vienna (BOKU)
Konrad-Lorenz-Str. 20, 3430 Tulln, Austria
e-mail: sebastian.michel@boku.ac.at

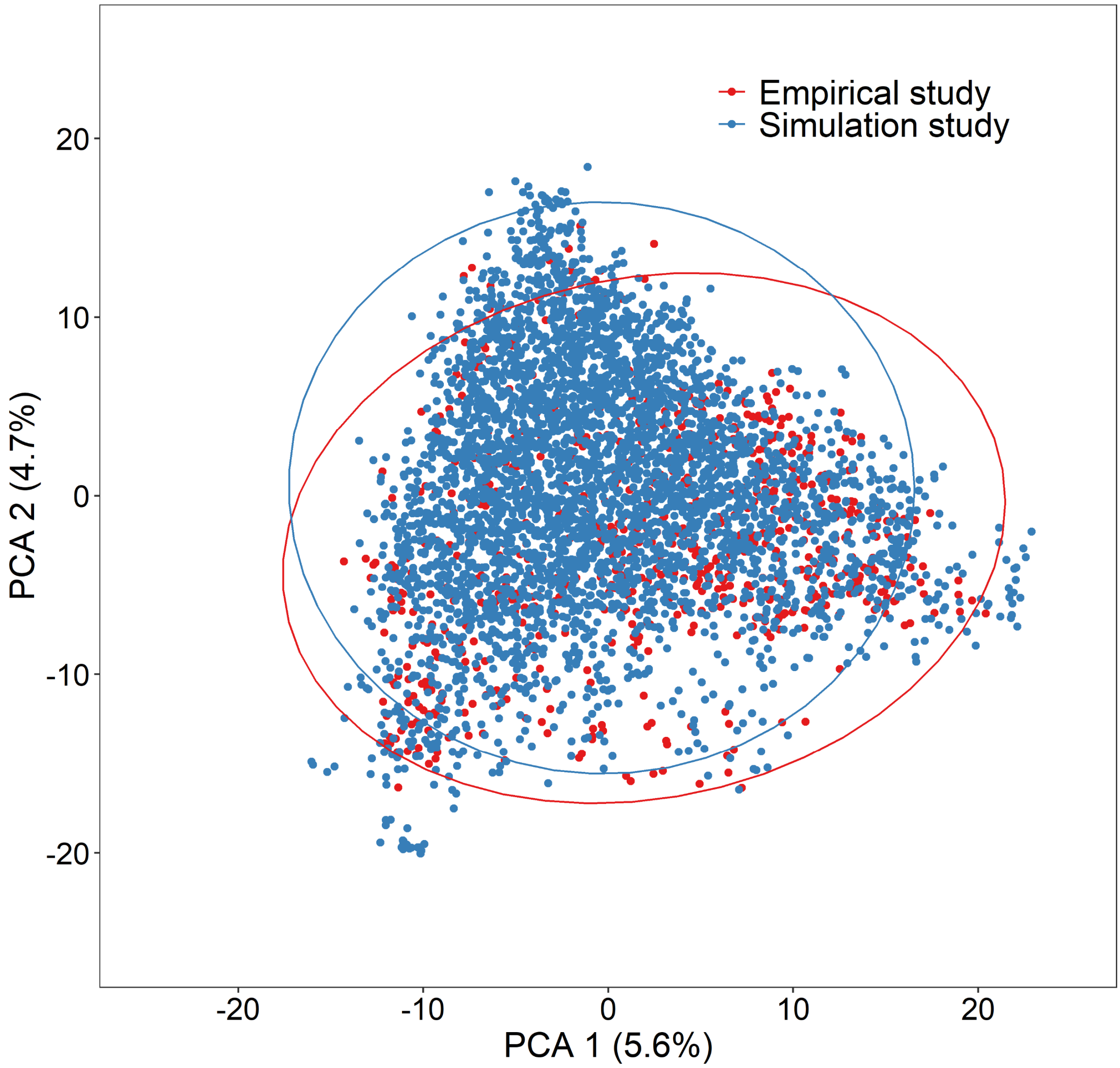

Figure S1 Principal component analysis of the 4577 lines involved in the study. A total of 4124 lines were used in the simulation study and 722 lines in the empirical study with 269 lines being part of both sets.

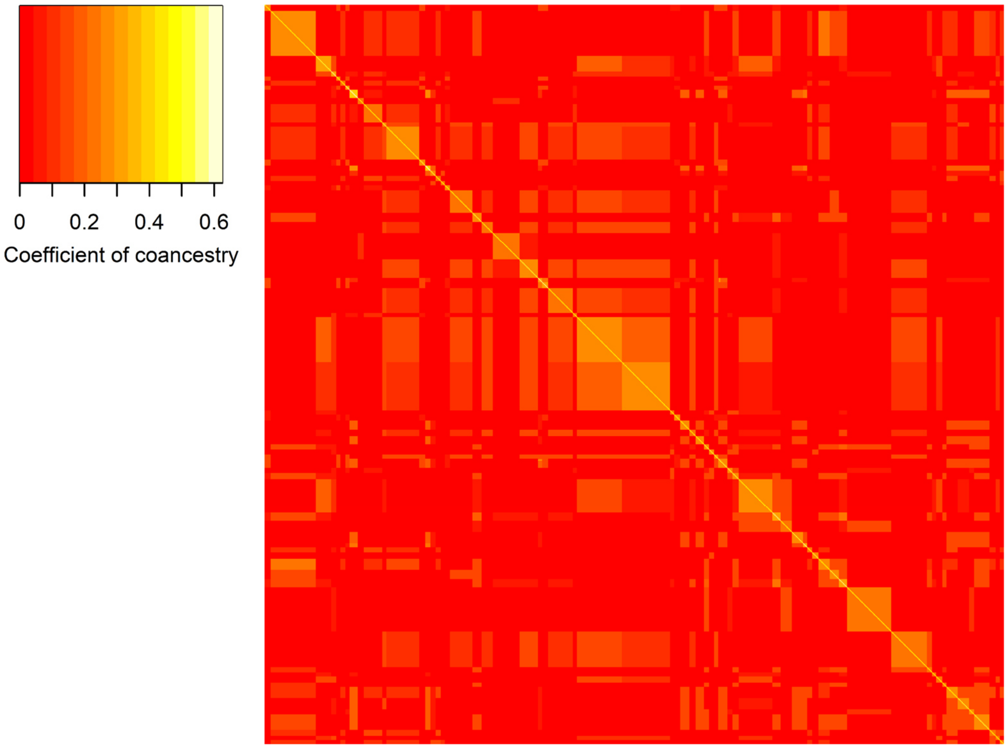

Figure S2 Heatmap of the coefficient of co-ancestry based on pedigree records of the 722 lines involved in the empirical study.

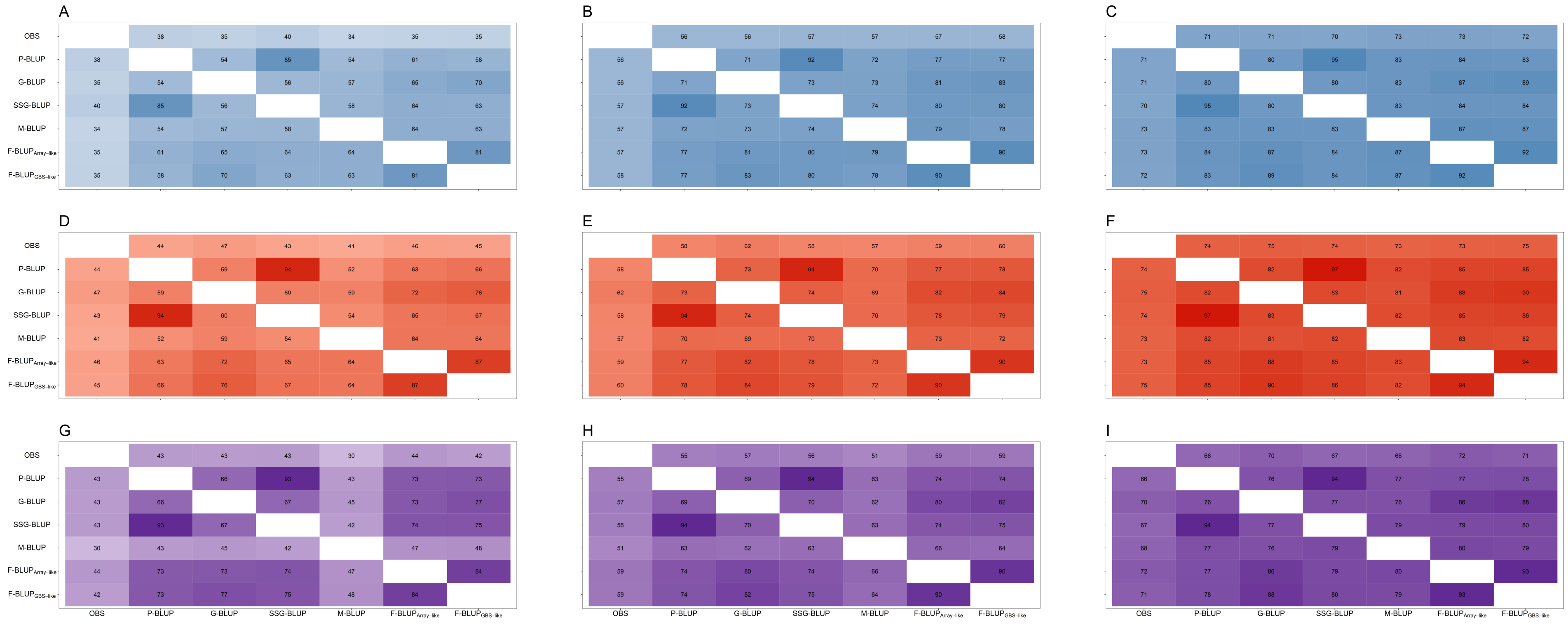

Figure S3 Heatmaps of the averaged overlap in percent for grain yield (A-C), protein content (D-F), and protein yield (G-I) of the best 20% (right column), 40% (centre column), and 60% (left column) of the lines selected by each of the tested prediction models. The phenotypic observations (OBS) were compared with prediction models using pedigree (P-BLUP) or genomic relationships from individual genotyped lines (G-BLUP) as well as a combined relationship matrix (SSG-BLUP), and genomic relationship matrices based on mid-parent (M-BLUP) or family bulk genotypes of the selection candidates with rounded (F-BLUP$_{\text{Array-like}}$) or unrounded (F-BLUP$_{\text{GBS-like}}$) average allele calls. Results are based on the 100 times replicated cross-validation scheme with the 63 families containing the 722 lines involved in the empirical study.

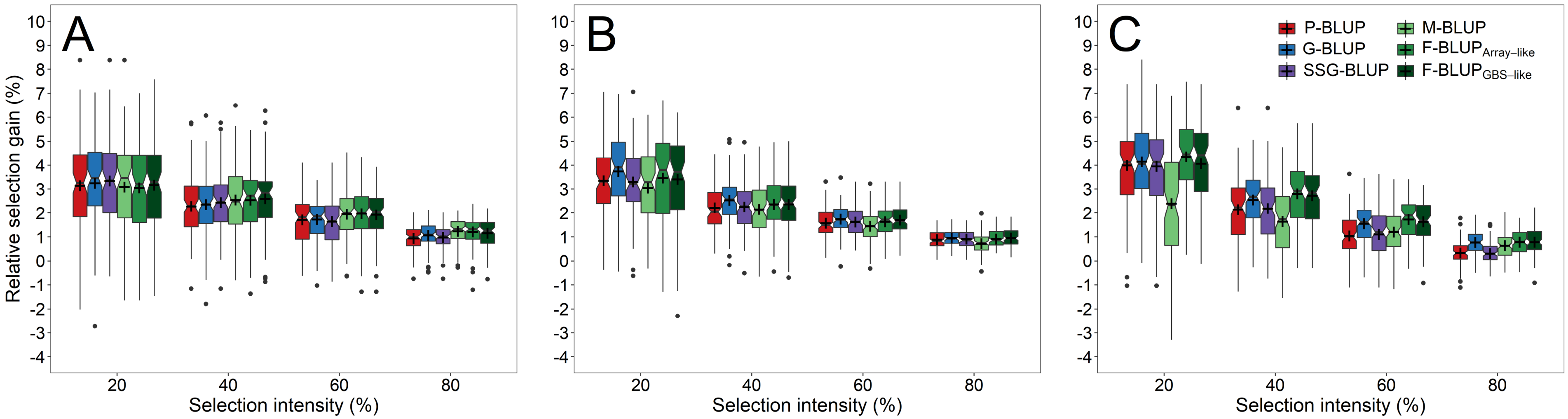

Figure S4 Boxplots of the relative selection gain for grain yield (A), protein content (B), and protein yield (C) of the best 20-80% selected lines based on prediction models using pedigree (P-BLUP) or genomic relationships from individual genotyped lines (G-BLUP) as well as a combined relationship matrix (SSG-BLUP) and genomic relationship matrices based on mid-parent (M-BLUP) or family bulk genotypes of the selection candidates with rounded ($F\text{-}BLUP_{Array\text{-}like}$) or unrounded ($F\text{-}BLUP_{GBS\text{-}like}$) average allele calls. Results are based on the 100 times replicated cross-validation scheme with the 63 families containing the 722 lines involved in the empirical study.

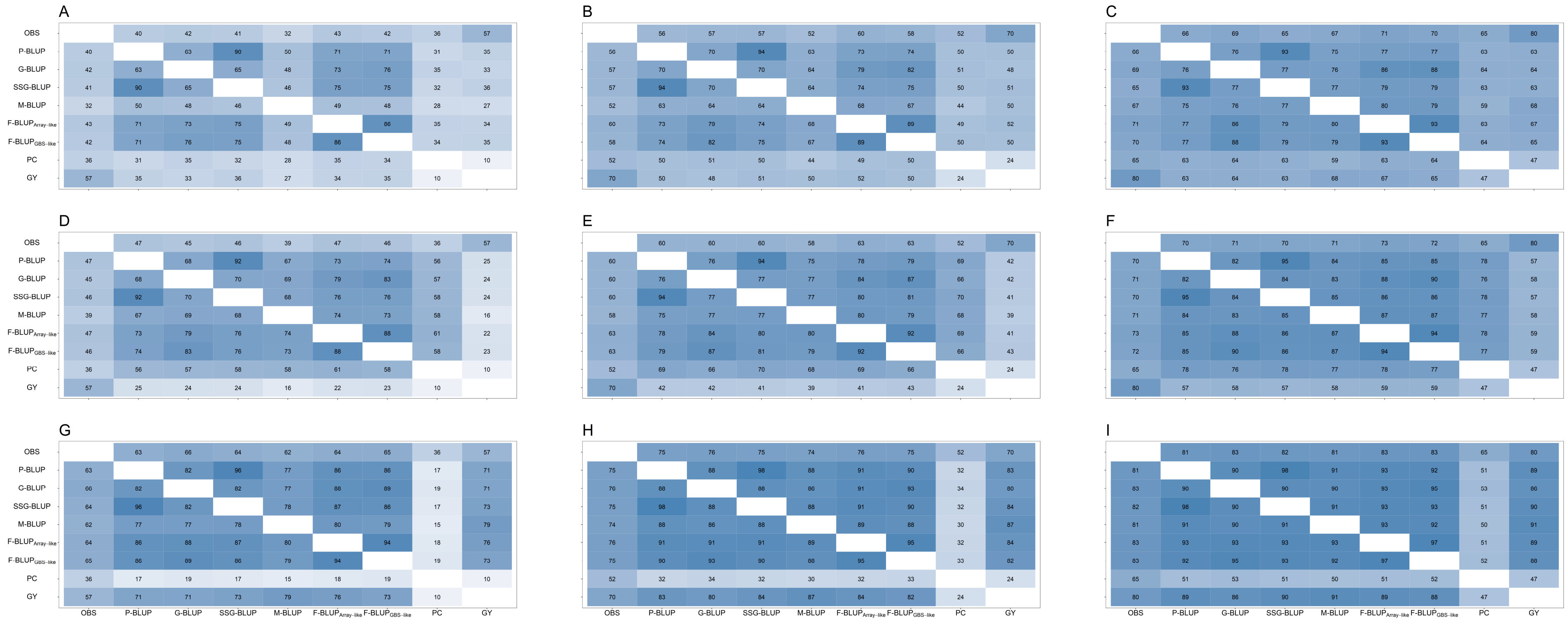

Figure S5 Heatmaps of the averaged overlap in percent of the best 20% (right column), 40% (centre column), and 60% (left column) of the lines when selecting for the protein yield by baseline prediction models without pre-existing information of the selection candidates (A-C) as well as trait-assisted prediction models with pre-existing information of the protein content (D-F) or grain yield (G-I). The phenotypic observations (OBS) were compared with the merit of an indirect selection by the protein content (PC) or grain yield (GY) as well as prediction models using pedigree (P-BLUP) or genomic relationships from individual genotyped lines (G-BLUP), a combined relationship matrix (SSG-BLUP), and genomic relationship matrices based on mid-parent (M-BLUP) or family bulk genotypes of the selection candidates with rounded ($F\text{-}BLUP_{Array\text{-}like}$) or unrounded ($F\text{-}BLUP_{GBS\text{-}like}$) average allele calls. Results are based on the 100 times replicated cross-validation scheme with the 63 families containing the 722 lines involved in the empirical study.

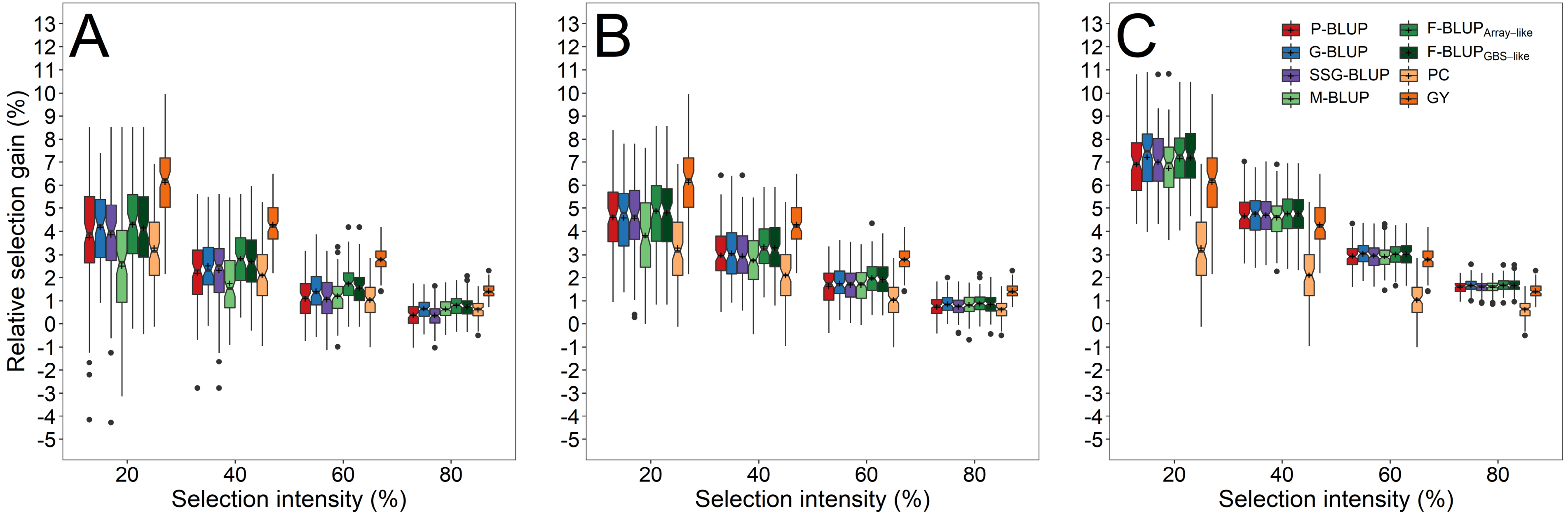

Figure S6 Boxplots of the relative selection gain for the protein content when selecting the best 20-80% of the lines by baseline prediction models without pre-existing information of the selection candidates (A) as well as trait-assisted prediction models with pre-existing information of the protein content (B) or grain yield (C). The merit of an indirect selection by the protein content (PC) or grain yield (GY) were compared with prediction models using pedigree (P-BLUP) or genomic relationships from individual genotyped lines (G-BLUP), a combined relationship matrix (SSG-BLUP), and genomic relationship matrices based on mid-parent (M-BLUP) or family bulk genotypes of the selection candidates with rounded (F-BLUP$_{Array-like}$) or unrounded (F-BLUP$_{GBS-like}$) average allele calls. Results are based on the 100 times replicated cross-validation scheme with the 63 families containing the 722 lines involved in the empirical study.

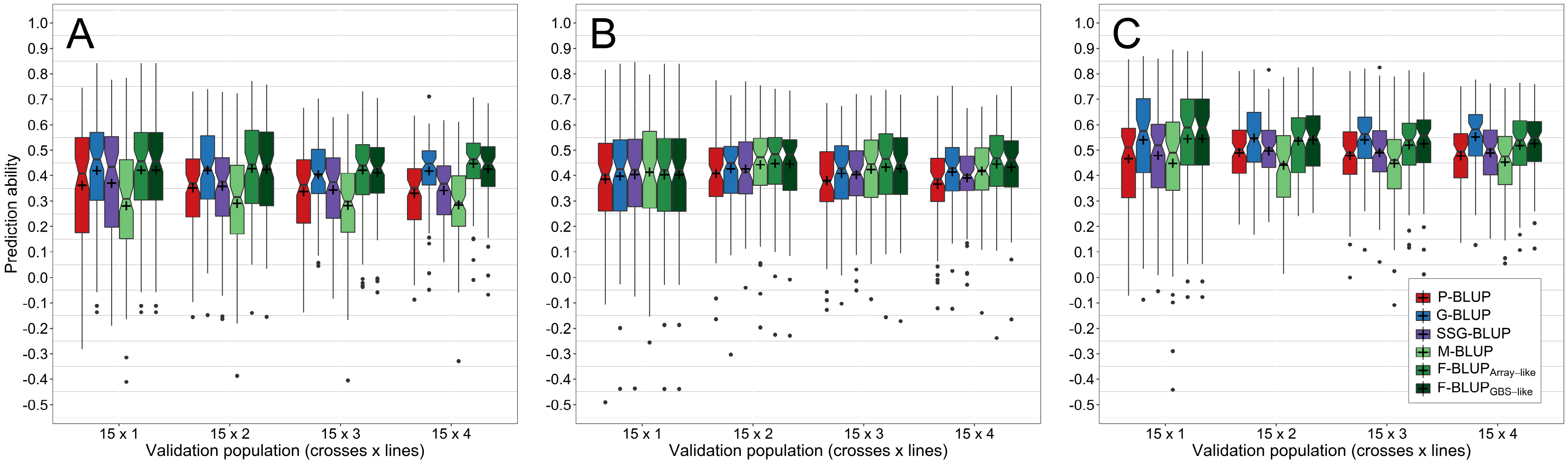

Figure S7 Boxplots for the prediction abilities for protein yield (A), grain yield (B), and protein content (C) with varying validation population sizes of one to four lines per family in the validation population when fitting prediction models with a pedigree (P-BLUP) or genomic relationships from individual genotyped lines (G-BLUP) as well as a combined relationship matrix (SSG-BLUP) and genomic relationship matrices based on mid-parent (M-BLUP) or family bulk genotypes of the selection candidates with rounded (F-BLUP$_{Array\text{-}like}$) or unrounded (F-BLUP$_{GBS\text{-}like}$) average allele calls. Results are based on the 100 times replicated cross-validation scheme with the 63 families containing the 722 lines involved in the empirical study.

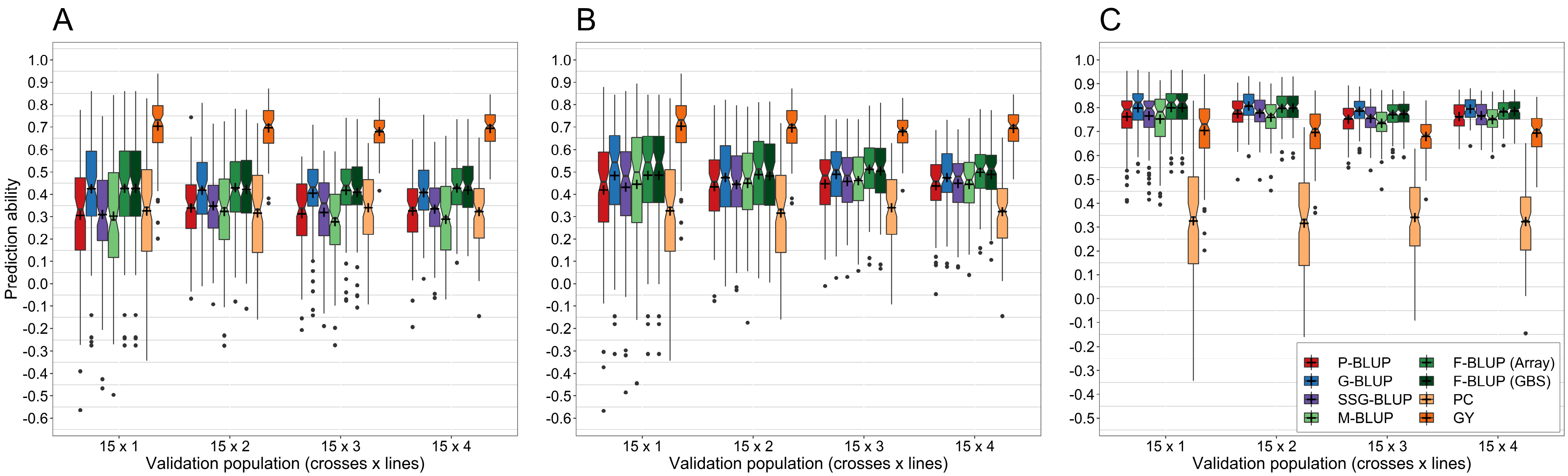

Figure S8 Boxplots of the prediction ability for protein yield with varying validation population sizes of one to four lines per family in the validation population when fitting prediction models with pedigree (P-BLUP) or genomic relationships from individual genotyped lines (G-BLUP) as well as a combined relationship matrix (SSG-BLUP) and genomic relationship matrices based on mid-parent (M-BLUP) or family bulk genotypes of the selection candidates with rounded (F-BLUP Array) or unrounded (F-BLUP GBS) average allele calls. The respective baseline models (A) were compared with a trait-assisted selection exploiting pre-existing information about the protein content (B) or grain yield (C) as well as with an indirect phenotypic prediction by the protein content (PC) or grain yield (GY).

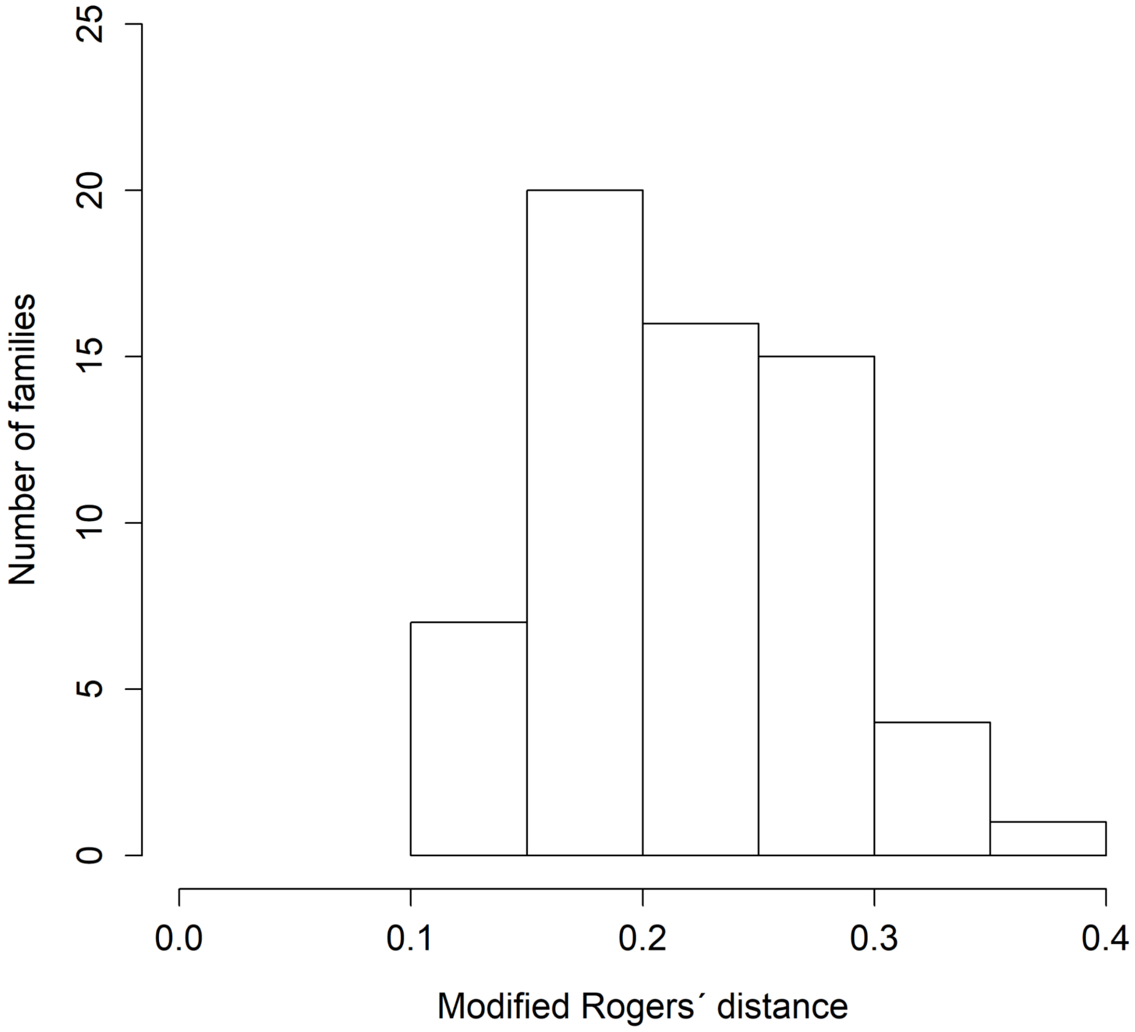

Figure S9 Modified Rogers´ distance between the mid-parent and the family bulk genotype of the 63 families involved in the empirical study. A Modified Rogers´ distance larger than zero implies a deviation of the observed family bulk genotype from its expectation i.e. the average genotype of both parents.

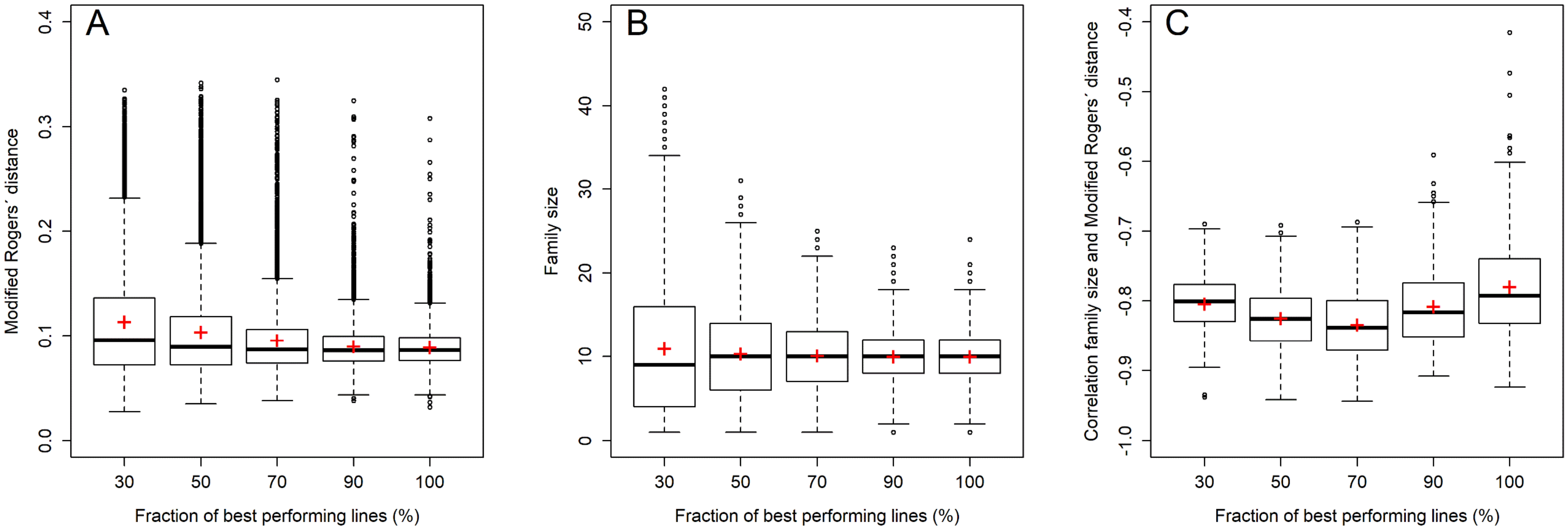

Figure S10 Modified Rogers´ distance, between the mid-parent and the family bulk genotype of the families involved in the simulation study (A) as well as their size (B) and the correlation between family size and Modified Rogers´ distance (C) for different fractions of pre-selected lines (validation scheme A). A Modified Rogers´ distance larger than zero implies a deviation of the observed family bulk genotype from its expectation i.e. the average genotype of both parents. A negative correlation between the Modified Rogers´ distance and the family size suggests furthermore that smaller families have a larger deviation from this expected genotype.

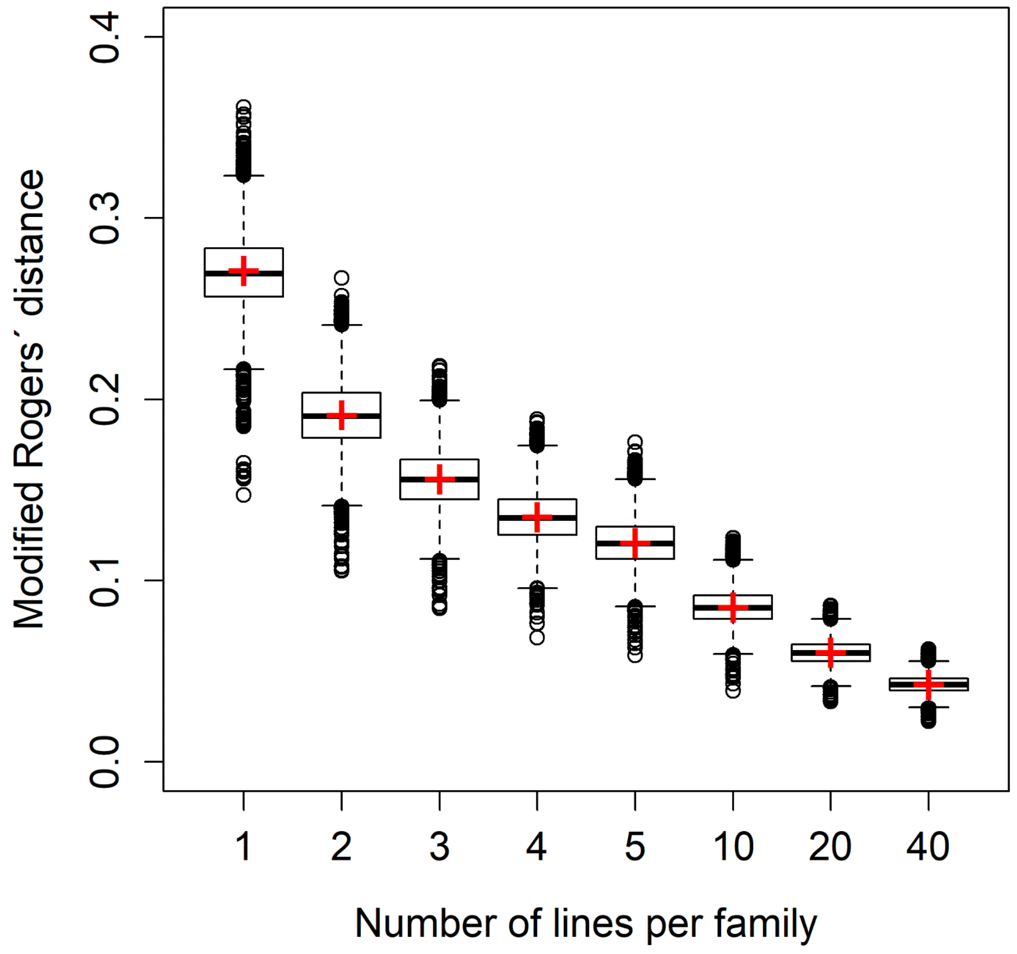

Figure S11 Modified Rogers´ distance, between the mid-parent and the family bulk genotype of the families involved in the simulation study with a varying number of randomly sampled lines from each of the families constituting the validation population (validation scheme B). A Modified Rogers´ distance larger than zero implies a deviation of the observed family bulk genotype from its expectation i.e. the average genotype of both parents.

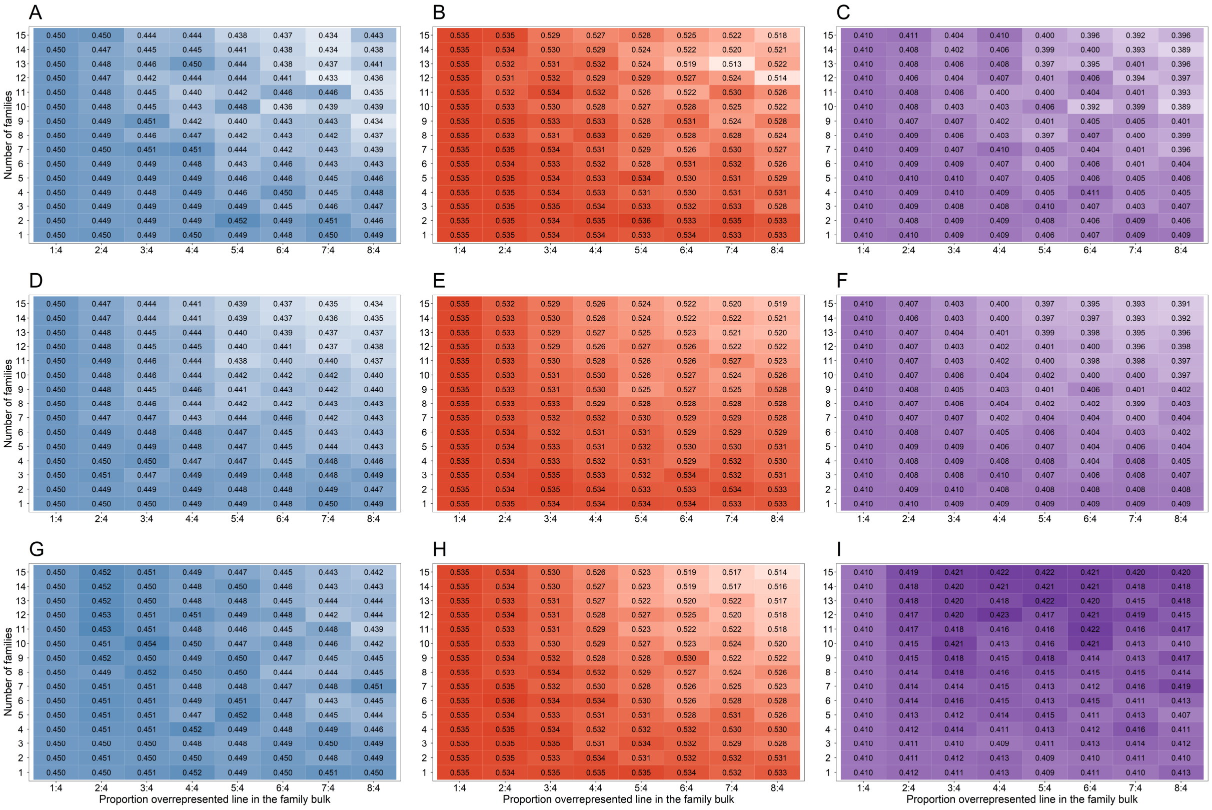

Figure S12 Heatmap of the prediction ability of the family bulk model with unrounded averaged allele calls ($\text{F-BLUP}_{\text{GBS-like}}$) for grain yield (left column), protein content (centre column), and protein yield (left column) when increasing the proportion of one overrepresented line in the bulk as well as increasing the number of families with an overrepresent line. Overrepresentation was facilitated sampling a random line from a family (A-C), the line that has the closest (D-F) or most distant (G-I) relationship to average of an equally represented bulk and increasing the frequency of the respective line from one to eight copies before averaging allele calls within each family bulk. Results are based on an 100 times replicated resampling scheme by randomly sampling 45 families and four lines per family into a training population, and 15 different families and four lines per family into a validation population as described in the main text of the manuscript. The proportion 1:4 is thus equivalent to a balanced sampling of all four lines within a given family bulk of the validation population.
